# Suppression of colitis by adoptive transfer of helminth antigen-treated dendritic cells requires interleukin-4 receptor-α signaling

**DOI:** 10.1038/srep40631

**Published:** 2017-01-17

**Authors:** C. E. Matisz, B. Faz-López, E. Thomson, A. Al Rajabi, F. Lopes, L. I. Terrazas, A. Wang, K. A. Sharkey, D. M. McKay

**Affiliations:** 1 Gastrointestinal Research Group and Inflammation Research Network, Department of Physiology and Pharmacology, Calvin, Joan and Phoebe Snyder Institute for Chronic Diseases, Cumming School of Medicine, University of Calgary, Canada; 2 Unidad de Biomedicina, FES Iztacala, Universidad Nacional Autónoma de México, Estado de México, México; 3 Hotchkiss Brain Institute, University of Calgary, Calgary, Alberta, Canada

## Abstract

Infection with helminth parasites has been explored as a treatment for autoimmune and inflammatory diseases. As helminth antigens have potent immunomodulation properties capable of inducing regulatory programs in a variety of cell types, transferring cells treated with helminth antigens represents a novel extension to helminth therapy. Previous work determined that transfer of bone marrow-derived dendritic cells (DC) pulsed with a crude extract of the tapeworm *Hymenolepis diminuta* (HD) can suppress colitis in recipient mice. The present study explored the mechanism of disease suppression and the importance of interleukin (IL)-4 signaling. Transfer of HD-DCs suppressed dinitrobenzene sulfonic acid (DNBS)-induced colitis through activation of recipient IL-4 receptor-α. The transferred HD-DCs required IL-4Rα and the capacity to secrete IL-10 to drive IL-4 and IL-10 production and to suppress colitis in recipient mice. Treatment of DCs with IL-4 evokes an alternatively activated phenotype, but adoptive transfer of these cells did not affect the outcome of colitis. Collectively, these studies demonstrate the complexity between IL-4 and IL-10 in donor cells and recipient, and the requirement for parasite- and host-derived factors in this novel form of cell therapy. Thus IL-4Rα signaling is revealed as a pathway that could be exploited for helminth antigen cell-based therapy.

Epidemiological studies that highlight the inverse relationship between exposure to helminth parasites and the incidence of inflammatory disease, combined with the known immunomodulatory capacity of helminths, have led to a renewed interest in the “therapeutic helminth”^1^,^2^,^3^. Analyses of murine models of disease, patient case reports, and small clinic trials have illustrated the suppression of inflammatory disease by infection with helminth parasites^4^,^5^,^6^,^7^,^8^. As an extension of this approach, systemic administration of antigens and secreted molecules from a variety of helminth parasites has been used to inhibit disease in rodent model systems^9^,^10^. A more recent aspect of helminth therapy is the prospect of using immune cells pulsed with or educated by exposure to helminth antigens^11^,^12^. Cellular immunotherapy, with hematopoietic or mesenchymal stem cells, is used to treat a variety of diseases^13^,^14^,^15^, and is being considered as an approach to treat inflammatory bowel disease, notably Crohn’s disease^16^,^17^,^18^,^19^.

We have recently shown that adoptive transfer of dendritic cells (DC) treated with a somatic extract of the adult rat tapeworm, *Hymenolepis diminuta* (HD) (HD-DCs), significantly attenuated the severity of di-nitrobenzene sulphonic acid (DNBS)-induced colitis in mice. The suppression of disease in the HD-DC treated mice was dependent on mobilization of adaptive immunity and was associated with the capacity of splenocytes in the recipients to produce IL-10 and IL-4^20^. While a role for IL-10 in the anti-colitic effect was defined, the significance, if any, of the increased production of IL-4 in the context of the biology of the DC was not addressed. Thus, this was the focus of the present study.

It has been shown that IL-4 converts DCs to an alternatively activated phenotype (AADC)^21^, somewhat analogous to the IL-4-induced alternatively activated macrophage (AAM)^22^, but little is known of the function of the former cell type. In the present study, using IL-4 receptor-α knock-out mice as donors or recipients of HD-DCs revealed that the anti-colitic effect of these cells required IL-4Rα on both the transferred cell and in the recipient mice. However, adoptive transfer of IL-4-induced AADC had no impact on the outcome of DNBS-induced colitis. Thus, the combination of exposure to an extract of *H. diminuta* and IL-4 generates a regulatory DC that can suppress colitis. These findings demonstrate the importance of IL-4Rα signaling in helminth antigen-pulsed DCs, and highlight a novel pathway that could be exploited for the use of helminth-antigen cell-based therapy.

## Results

### Attenuation of colitis by HD-DCs is positively correlated with splenic IL-4

**Figure 1:**
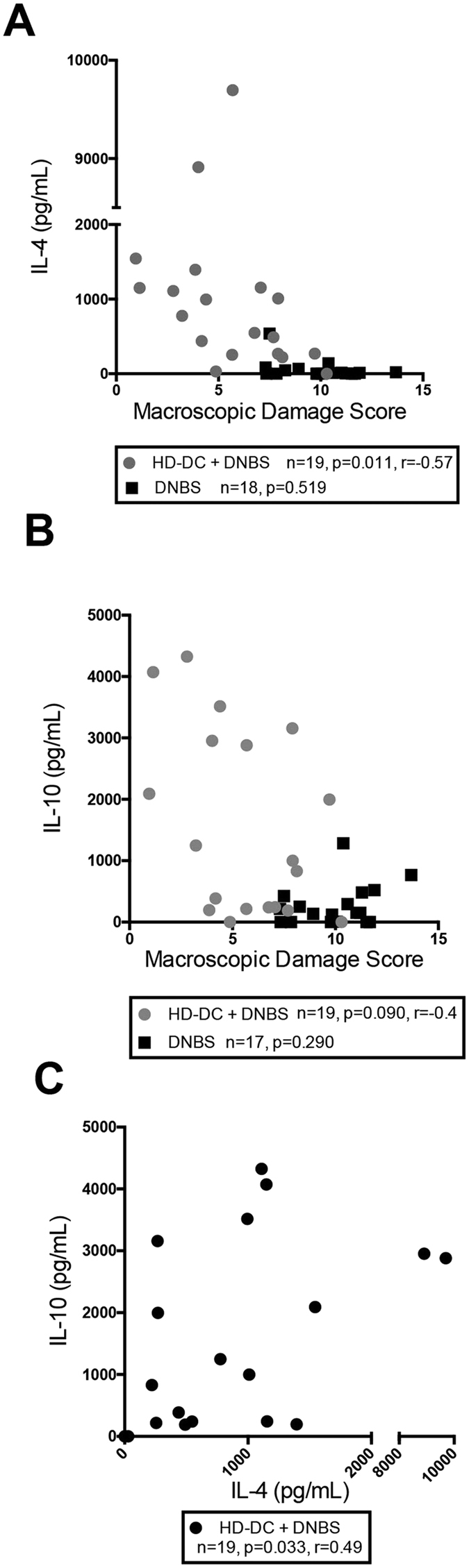
Suppression of colitis by HD-DC transfer is correlated with splenic IL-4 and IL-10 production. Individual macroscopic disease scores of mice challenged with DNBS, some of which had received 10^6^ HD-DCs i.p. 48 h prior, were plotted alongside their levels of splenic IL-4 (**A**) IL-10 (**B**). Splenic IL-4 and IL-10 in recipients of HD-DCs were plotted against each other in (**C**). Cytokine levels were measured by ELISA from the supernatants of conA (2 μg/mL) stimulated splenocytes (5 × 10^6^/mL). As a D’Agonstino & Pearson omnibus normality test determined cytokine data was not normally distributed; Spearman correlation test was performed for all graphs. Data from 4 independent experiments.

Adoptive transfer of HD-DCs attenuates the severity of DNBS-induced colitis and this is accompanied by increased production of IL-4 by mitogen-stimulated splenocytes from the recipient mice^20^. Correlation analysis revealed a significant relationship between splenocyte IL-4 production and colitis disease activity score, such that those mice treated with HD-DCs that produced more IL-4 had less severe colitis (Fig. 1A). A trend towards a negative correlation between IL-10 and disease severity (Fig. 1B), and a positive correlation between splenic IL-4 and IL-10 production (Fig. 1C) was also observed. Thus, a role for IL-4 in the anti-colitic effect conferred by adoptive transfer of HD-DCs was tested.

### Recipients require IL-4Ra signaling for HD-DC suppression of colitis

**Figure 2:**
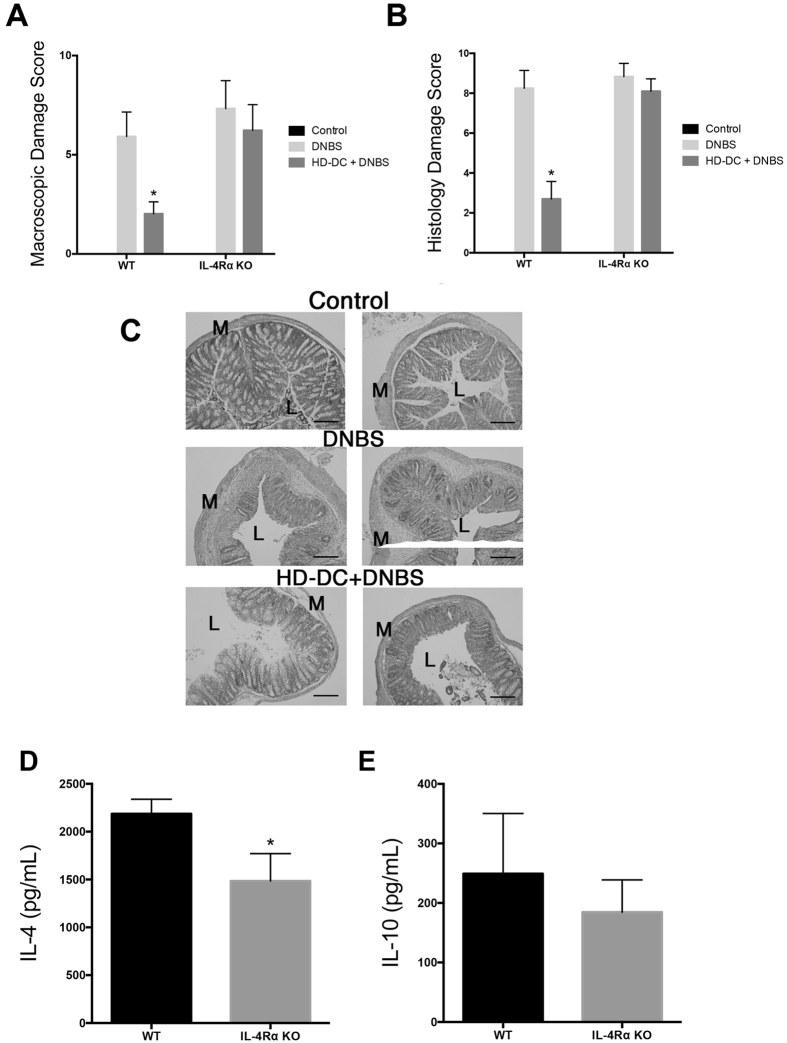
HD-DCs do not suppress DNBS in IL-4Rα^−^/^−^ mice. In-house bred wild-type (WT) or IL-4Rα^−/−^ on a Balb/c background were given 10^6^ HD-DC ip. 48 h prior to DNBS (3 mg, i.r.) challenge. Mice were necropsied and assessed 72 h later via (**A**) macroscopic damage score and (**B**) histological damage score; (**C**) representative micrographs of H&E stained cross-sections of colonic tissue: L; lumen; M: muscle; scale bar 300 μm. Splenocytes were isolated (5 × 10^6^), stimulated with conA (2 μg/mL, 48 h) and supernatants examined for levels of IL-4 (**D**) and IL-10 (**E)** by ELISA. Data are shown as mean ± SEM; **A–C** are representative from one of two independent experiments, (*p ≤ 0.05 vs. DNBS group, Kruskal-Wallis with Dunn’s post-test, n = 3–6 mice/group) and **D,E** are pooled from two independent experiments (n = 7–9; two-tailed unpaired Student’s t-test).

Adoptive transfer of wild-type (WT) HD-DCs into normal mice significantly suppressed DNBS-induced colitis, as before^20^, but this was not observed in recipient IL-4Rα^−/−^ mice (Fig. 2A–C). In accordance with these data, splenocytes from WT mice given HD-DCs challenged with DNBS showed increased IL-4 and IL-10 output from stimulated splenocytes compared to time-matched IL-4Rα^−/−^ mice (Fig. 2D,E). Administration of HD-DCs to IL-4Rα^−/−^ mice that did not get DNBS also resulted in less IL-4 output from splenic T cells stimulated 5 days after receiving the cells compared to WT mice [Mean ± SEM of IL-4: WT recipients = 2188 ± 87 pg/mL; IL4Rα^−/−^ recipients = 1485 ± 165 pg/mL; p = 0.0198, n = 3]. Thus, an IL-4 feedback loop exists that is important for the anti-colitic effect of HD-DCs, such that these cells stimulate IL-4 production, which is important for the anti-colitic effect since the HD-DCs are ineffective in IL-4Rα^−/−^ mice (Fig. 2A–C).

### HD-DCs require IL-4Ra to suppress DNBS colitis in recipients

**Figure 3:**
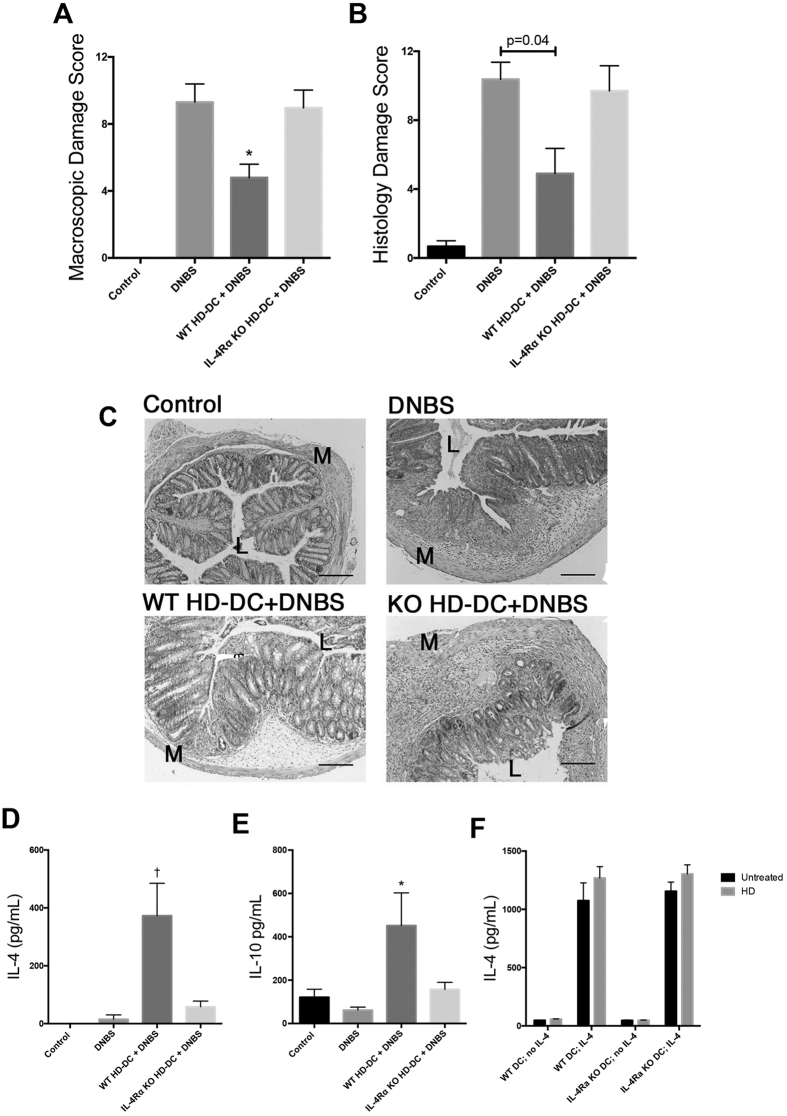
HD-DCs require IL-4Rα to suppress DNBS colitis in recipients. Balb/c mice were given 10^6^ wild-type or IL-4Rα^−/−^ HD-DC ip. 48 h prior to DNBS (3 mg, ir.) challenge. Mice were necropsied and assessed 72 h later via (**A**) macroscopic damage score (Kruskal Wallis with Dunn’s post-test *n* = 6–10 mice/group from 2 experiments) and (**B**) histological damage score (Mann-Whitney *n* = 6–10 mice/group from 2 experiments); (**C**) representative micrographs of H&E stained cross-sections of colonic tissue: L; lumen; M: muscle; scale bar 300 μm. (**D,E**) Splenocytes were isolated (5 × 10^6^), stimulated with conA (2 μg/mL, 48 h) and supernatants examined for levels of IL-4 and IL-10 (*n* = 6–10 mice/group from 2 experiments) by ELISA, and analyzed by one-way ANOVA with Tukey’s post-test. (**F**) WT and IL-4Rα^−/−^ DCs were treated with HD antigen (100 μg/mL) ± IL-4 (20 ng/mL); n = 4 from 1 experiment; one-way ANOVA with Sidak’s multiple comparison among selected groups. All data are shown as mean ± SEM; p ≤ 0.05 *compared to DNBS, †compared to all groups.

Considering the possibility that IL-4 signaling in the dendritic cell could be important for its anti-colitic activity, WT HD-DCs and IL-4Rα^−/−^ HD-DCs were transferred to WT mice, 48 hours prior to DNBS challenge. Mice that received WT HD-DC, but not IL-4Rα^−/−^ HD-DC, displayed significantly attenuated macroscopic damage (Fig. 3A), and reduced histological damage (Fig. 3B,C). The absence of IL-4Rα completely abrogated the ability of HD-DCs to drive splenic IL-4 and IL-10 in recipients (Fig. 3D,E). It has been reported that IL-4 can induce IL-4 production by DCs^23^. However, treatment with IL-4 resulted in similar levels of IL-4 in the supernatants of WT and IL4Rα^−/−^ DCs, and co-treatment with HD antigen did not significantly enhance the ability of IL-4 to induce DC IL-4 synthesis (Fig. 3F). In addition, IL-4 mRNA was not detected in DCs treated with HD antigen, IL-4, or in combination.

### HD-DCs induce a population of IL-4 producing CD4^+^ and CD19^+^ splenocytes

**Figure 4:**
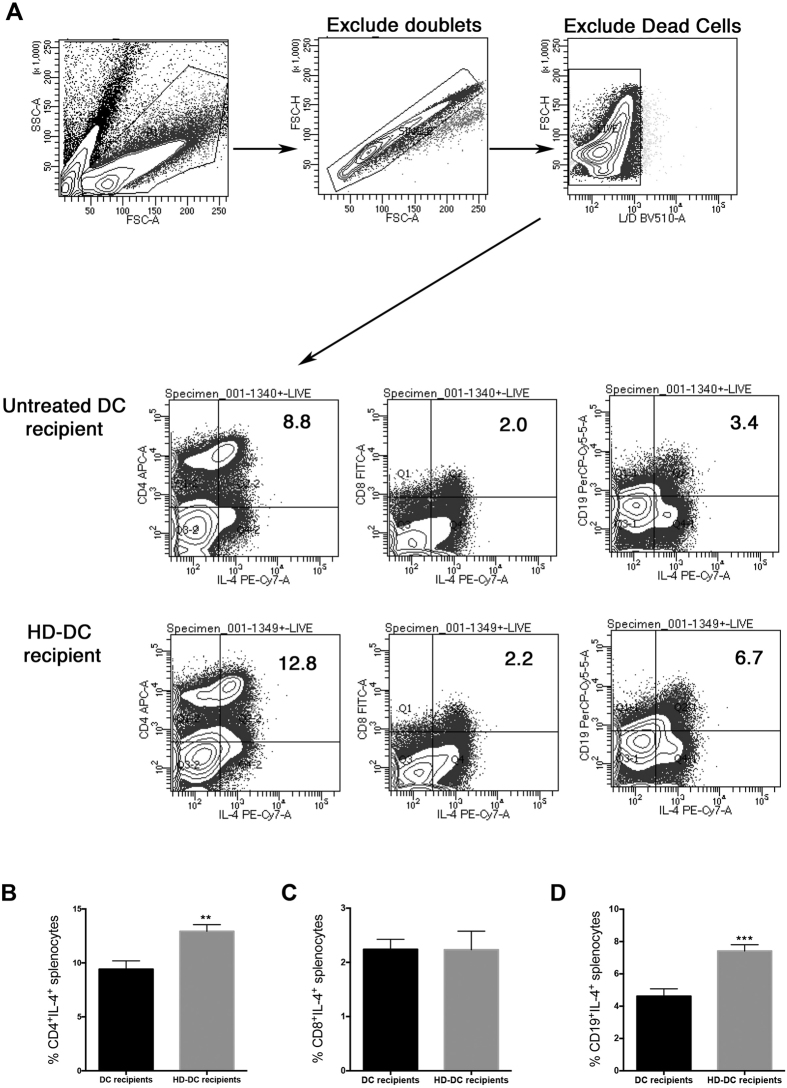
HD-DCs induce IL-4 producing CD4^+^ and CD19^+^ splenocytes in recipient mice. Untreated DC or HD-DCs (10^6^) were adoptively transferred into Balb/c mice. Five days later, total splenocytes were counted, and following fixation and permeabilization, splenocytes were stained and analyzed by flow cytometry. Complete gating strategy is depicted in (**A**). The proportion of IL-4 positive cells was assessed in CD4^+^ (**B**), CD8^+^ (**C**), and CD19^+^ (**D**) splenocytes. All data are shown as mean ± SEM; n = 5–6 from one of two representative experiments; **p ≤ 0.01, ***p ≤ 0.001.; unpaired two-tailed Student’s t-test.

To assess the source of splenic IL-4, mice were administered DCs or HD-DCs, and 5 days later splenocytes were counted and stained for IL-4 (Fig. 4A). Adoptive transfer of HD-DC significantly increased the proportion of IL-4 secreting CD4^+^ and CD19^+^ splenocytes in recipient mice (Fig. 4B,D). No differences in CD8^+^IL-4^+^ cells were observed (Fig. 4C).

### IL-10^−/−^ HD-DCs do not suppress DNBS colitis

**Figure 5:**
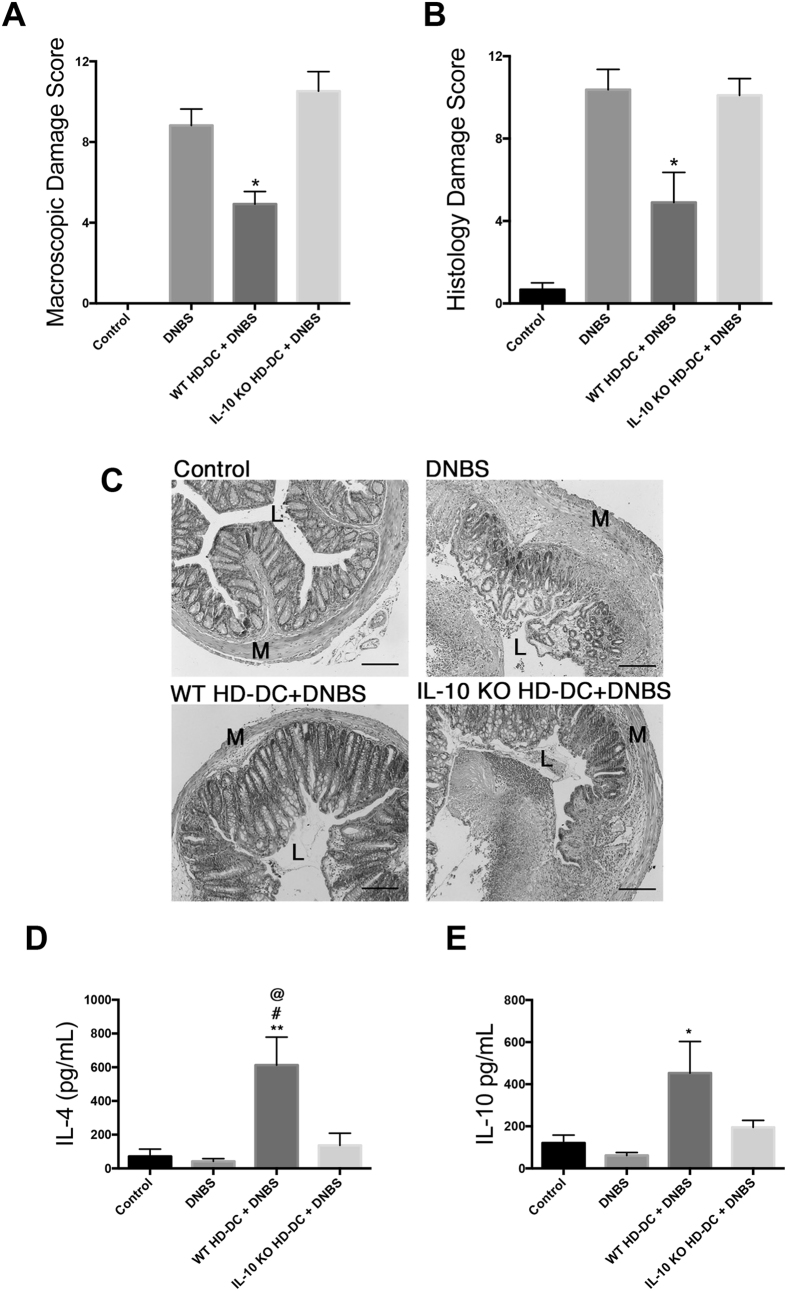
IL-10 KO HD-DCs cannot suppress colitis. Balb/c mice were given 10^6^ wild-type or IL-10^−/−^ HD-DC i.p. 48 h prior to DNBS (3 mg, i.r.) challenge. Mice were necropsied and assessed 72 h later via (**A**) macroscopic damage score and (**B**) histological damage score using Kruskal-Wallis with Dunn’s post-test. (**C**) Representative micrographs of H&E stained cross-sections of colonic tissue: L; lumen; M: muscle; scale bar 300 μm. Splenocytes were isolated (5 × 10^6^), stimulated with conA (2 μg/mL, 48 h) and supernatants examined for levels of IL-4 (**D**) and IL-10 (**E**) by ELISA, and analyzed by one-way ANOVA with Tukey’s post-test. Data are shown as mean ± SEM and are pooled from two independent experiments; n = 6–10 mice/group; *p ≤ 0.05, **p ≤ 0.01 compared to DNBS, # compared to control, @ compared to IL-10 KO HD-DCs.

IL-10 producing DCs have been demonstrated to drive IL-4 production in CD4^+^ cells *in vitro*^24^, and induce tolerogenic T cell populations^25^. Thus the importance of IL-10 secretion by adoptively transferred HD-DCs was considered. WT and IL-10^−/−^ HD-DCs were transferred to WT mice, 48 h prior to DNBS challenge. Contrary to WT HD-DCs, IL-10^−/−^ HD-DCs were unable to attenuate the severity of colitis (Fig. 5A–C), nor were they able to drive splenic IL-4 or IL-10 in recipients (Fig. 5D,E). Levels of transcript and secreted IL-10 were below level of detection in HD-DC treated cells *in vitro.*

### IL-4 alternatively activated DCs do not suppress DNBS-induced colitis

**Figure 6:**
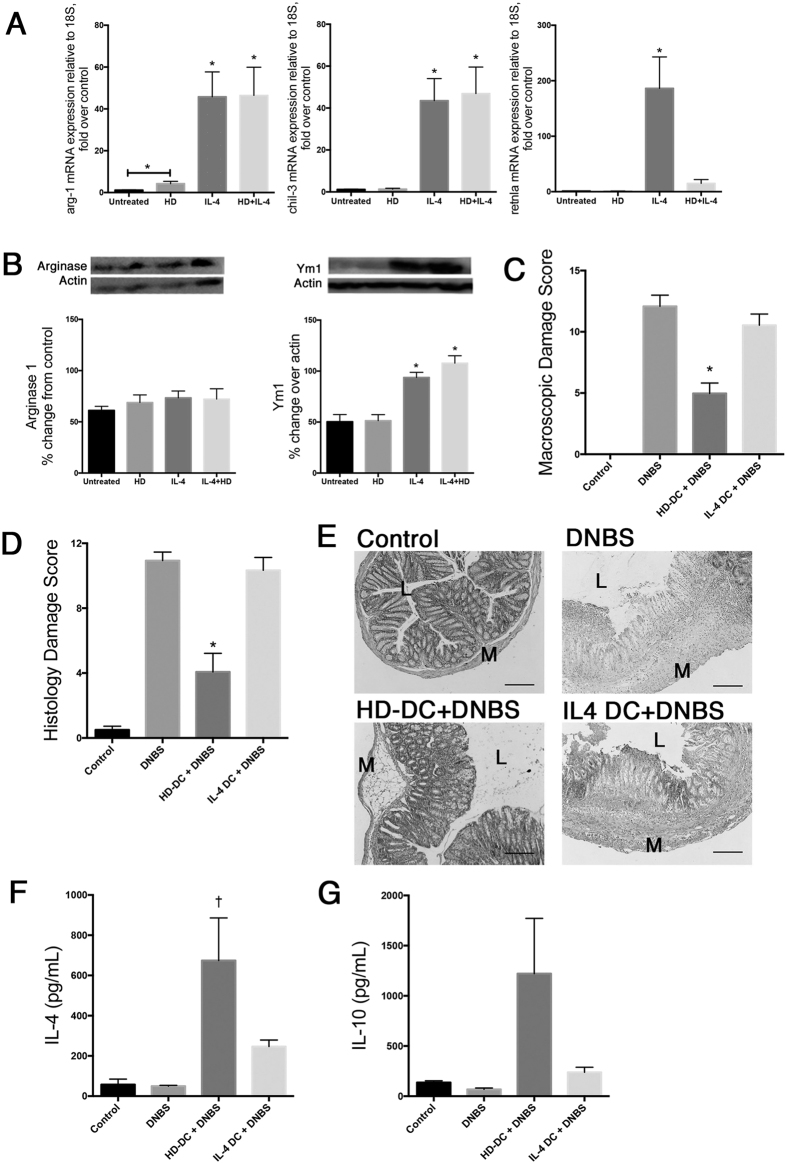
IL-4 alternatively activated DCs are not able to suppress colitis in recipients. Levels of arg-1, chil3 (Ym1), and retnla mRNA expression (**A**; n = 6–10 from 3 experiments) and arginase-1 and Ym1 protein expression (**B**; n = 6 from 2 experiments), with representative western blots, in untreated DCs, and DCs treated with HD antigen (100 μg/mL) and/or IL-4 (20 ng/mL) for 24 h. Data are shown as mean ± SEM; Repeated one-way ANOVA with Dunn’s post-test; *p ≤ 0.05. For additional experiments, Balb/c mice were given 10^6^ HD-DC (100 μg/mL) or IL-4 alternatively activated (AA) DCs (20 ng/mL) ip. 48 h prior to DNBS (3 mg, i.r.) challenge. Mice were necropsied and assessed 72 h later via (**C**) macroscopic damage score and (**D**) histological damage score, with Kruskal-Wallis and Dunn’s post-test. (**E**) Representative micrographs of H&E stained cross-sections of colonic tissue: L; lumen; M: muscle; scale bar 300 μm). Splenocytes were isolated (5 × 10^6^), stimulated with conA (2 μg/mL, 48 h) and supernatants examined for levels of IL-4 (**F**) and IL-10 (**G**) by ELISA, and analyzed by one-way ANOVA with Tukey’s post-test. Data are shown as mean ± SEM and are pooled from two independent experiments; n = 6–8 mice/group; p < 0.05: *compared to DNBS, †compared to all groups.

IL-4 treated macrophages take on an alternatively activated phenotype (AAMs) characterized by expression of arginase1, Ym1, and Relm-α^22^, and the adoptive transfer of IL-4 AAMs can suppress DNBS-induced colitis^26^. Similarly, analysis of Ym1 and Relm-α expression reveals that IL-4 causes alternative activation in DCs (AADCs)^21^, raising the possibility that HD antigen was, through its induction of IL-4, inducing an AADC phenotype that could block colitis. Replicating the findings of Cook *et al*., IL-4 increased Relm-α transcript and Ym1 and transcript (i.e. chil-3) and protein expression in DC, and we find that arginase-1 mRNA and protein are also up-regulated (Fig. 6A,B). HD antigen alone did not affect the expression of any of these markers, nor did it modulate the effect of IL-4 with the exception of suppression of Relm-α mRNA expression (Fig. 6A,B).

In contrast to HD-DCs, adoptive transfer of IL-4 AADCs did not affect the outcome of DNBS-induced colitis as assessed by macroscopic and histopathology damage scores (Fig. 6C–E), and did not induce IL-4 or IL-10 in the spleen (Fig. 6F,G). Adoptive transfer of DCs treated with HD+IL-4 attenuated DNBS colitis by ~35%, a degree of suppression similar to ~41% observed with HD-DCs as assessed by disease activity score (DNBS = 13.7 ± 0.9, n = 3; HD-DC+DNBS = 8.2 ± 3.1, n = 3; HD+IL-4-DC+DNBS = 9.0 ± 1.0, n = 4; mean ± SEM).

### HD modulates IL-4 enhanced LPS pro-inflammatory responses

**Figure 7:**
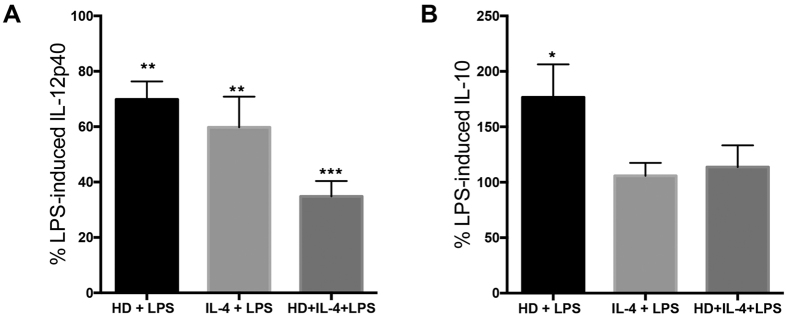
IL-12 and IL-10 production by HD-DCs exposed to LPS and IL-4. Dendritic cells were treated with HD antigen (100 μg/mL) 24 h prior to treatment with IL-4 (20 ng/mL) and LPS (1 μg/mL). Levels of IL-12p40 and IL-10 were assessed in the supernatants by ELISA 24 hours post stimulation of BMDCs with LPS and depicted as proportions of LPS-induced IL-12 (**A**; n = 8 from 2 experiments) and IL-10 (**B**; n = 4 from 1 experiment). Data are shown as mean ± SEM; one-sample, two-tailed t-test to hypothetical mean of 100; *p ≤ 0.05; **p ≤ 0.01, ***p ≤ 0.001.

IL-4 not only elicits an AADC phenotype, it can also promote pro-inflammatory responses in DCs *in vitro*^21^,^27^. Since TNBS-induced colitis is accompanied by increased serum levels of LPS^28^, it is likely that transferred cells are exposed to LPS in DNBS challenged mice. To mimic this putative *in vivo* interaction, DCs were treated with HD antigen for 24 h, and then stimulated with IL-4 and LPS for an additional 24 h. Levels of IL-12 (p40) and IL-10 produced by DCs treated with IL-4, HD antigen alone, or in combination were below detection by ELISA. Treatment with HD antigen significantly suppressed LPS-induced IL-12 production by approximately 30%, while DCs treated with HD and IL-4 exhibited a 65% reduction in LPS-induced IL-12 (Fig. 7A; LPS 4.6 ± 0.8; HD+LPS 3.3 ± 0.7; IL-4+LPS 2.7 ± 0.6; HD+IL-4+LPS 1.6 ± 0.3 IL-12p40 ng/mL). Only DCs treated with HD antigen in the absence of IL-4 displayed significant increases (175%) in LPS-induced IL-10 (Fig. 7B; LPS 250 ± 11; HD+LPS 443 ± 46; IL-4+LPS 264 ± 13; HD+IL-4+LPS 283 ± 16 IL-10 pg/mL). IL-4 enhanced LPS-induced IL-12 production only when added 24 h prior to LPS challenge, and this too was attenuated by HD (mean ± SEM of IL-12p40: LPS = 2484 ± 449; LPS+IL-4 = 5310 ± 1106*; HD+LPS = 1758 ± 487; HD+IL-4+LPS = 2611 ± 731 pg/mL; p < 0.05; n = 11).

## Discussion

Helminth therapy has been presented as a novel and natural way to inhibit auto-inflammatory diseases^29^. These are heterogeneous disorders and so it is reasonable to speculate that effective helminth therapy would be equally heterogeneous, requiring a specific helminth, a helminth extract or purified bioactive molecule, or autologous transplantation using the patients’ own cells pulsed with a specific helminth extract: these approaches would mobilize a diverse array of anti-inflammatory mechanisms^30^. Adoptive transfer of HD-DCs suppresses DNBS-induced colitis, with the greatest benefit occurring in those mice showing significant increases in IL-10 and IL-4 production by splenic T cells^20^. Interleukin-10 is a prototypic anti-inflammatory cytokine and having defined its involvement in HD-DC-suppression of colitis^20^; we focused on the putative role of IL-4. The data obtained indicate that IL-4Rα – and by inference IL-4 signaling – is required in both recipient and donor for HD-DCs to block DNBS-induced colitis.

Infection with helminth parasites elicits IL-4 production in mammalian hosts from a variety of cells^31^,^32^,^33^, with T cells activated by helminth-educated DCs being a major source of the cytokine^34^,^35^,^36^. For instance, mice infected with *H. diminuta* have increased synthesis of IL-4^37^,^38^, and the demonstration that adoptive transfer of HD-DCs promotes splenocyte production of IL-4 upon reactivation *in vitro* is in accordance with the data from use of the parasite. Both CD4^+^ T cells and CD19^+^ B cells were identified as sources of IL-4 in mice receiving HD-DCs: the latter being noteworthy given the demonstration of an anti-colitic regulatory B cell in *H. diminuta*-infected mice^39^, and generation of an IL-4-producing B cell that was dependent on T cells and IL-4Rα in mice infected with the nematode parasite *Heligosoimoides polygyrus*^40^. Furthermore, the observation that functional IL-4Rα in recipient mice is required for the anti-colitic effect of HD-DCs is consistent with data showing mice lacking signal transducer and activator of transcription (STAT)-6, the major intracellular signaling pathway from the IL-4 receptor, are not protected from DNBS-induced colitis by infection with *H. diminuta*^7^.

A number of possibilities exist for the mechanism of action of IL-4Rα signaling in the suppression of colitis following adoptive transfer of HD-DCs. IL-4 is the prototypic TH2 cytokine perpetuating TH2 immunity^41^,^42^. It can exert anti-colitic effects, as shown by adenoviral delivery or liposome encapsulated plasmid DNA of IL-4 as a transgene suppression of TNBS-induced colitis^43^,^44^, or it can be pro-inflammatory with respect to allergic disorders^45^.

IL-4 will impede the development of TH1-dominated disease and TNBS/DNBS is often considered in this category^46^,^47^. In this context, IL-4 was found to suppress LPS-induced IL-12p40 production by DCs, an outcome enhanced in cells treated with HD antigen. Indeed, HD antigen evoked DC production of IL-10 when coupled with LPS exposure; an inability for DCs to produce IL-10 abrogated the capacity of the worm antigen to inhibit LPS-evoked IL-12p40 (unpublished observation). Although IL-4 is reported to drive pro-inflammatory responses in some contexts, we note that IL-4 only significantly exacerbated LPS-induced IL-12 when added prior to LPS challenge; this was significantly attenuated by HD antigen. In addition, IL-4 and IL-10 are often functionally coupled, underscoring the finding that adoptive transfer of HD-DCs invariably increased IL-4 and IL-10 output from the recipient’s splenocytes. Similarly, transfer of DCs pulsed with schistosome egg antigen resulted in up-regulation of IL-4 and IL-10, with IL-10 synthesis being IL-4Rα-dependent^21^. In addition, the expansion of IL-10^+^CD4^+^ T cells in *Nippostrongylus brasiliensis*-infected mice is significantly impaired in mice lacking the IL-4Rα, and this ultimately prevented the establishment of IL-4 responses^48^. Alternatively, IL-10 treated DCs induced IL-4 production in CD4^+^ cells^24^.

IL-4 promotes the production and secretion of mucus^31^ that protects the gut from irritants in the lumen and this is likely a component of the anti-colitic effects mediated by this cytokine. Finally, and directly linked to infection with helminth parasites and hence HD-DCs, IL-4 drives the development of alternatively activated macrophages (AAMs or M(IL-4)) that have been implicated in wound healing^49^ and the suppression of colitis-induced by DNBS, oxazolone and dextran sodium-sulfate (DSS)^26^,^50^,^51^.

Intriguingly, IL-4-treated DCs adopt an alternatively activated phenotype (AADC) reminiscent of the AAM, as characterized by increased expression of Ym1 and Relm-α^21^. We confirmed these findings and also noted up-regulation of arginase-1, which has been implicated in the suppression of colitis^52^,^53^ and is important in tissue repair after injury^54^,^55^. Comparison with AAMs predicts an anti-colitic ability of AADCs; however, this was not observed when IL-4 evoked AADCs were compared directly with HD-DCs in the DNBS model of colitis. These data imply that HD antigen elicits a unique regulatory phenotype, a speculation supported by data showing that HD-DCs displayed no increases in Ym1 or Relm-α mRNA and only small increases in arginase-1 mRNA, and HD antigen actually inhibited IL-4 induction of Relm-α mRNA in DCs. In accordance with these findings, a variety of helminth-extracts have been shown to affect the behavior of DCs and modify their response to pathogen-associated molecular patterns (see above)^10^,^56^,^57^.

Since transfer of HD-DCs evokes IL-4 production, and IL-4 as a paracrine or autocrine factor can direct DC function^21^ (while noting that HD antigen did not evoke DC synthesis of IL-4), this raised the possibility that at least part of the role of IL-4 in mice given HD-DCs could be to influence the fate of the transferred DC. This would appear to be the case, as adoptive transfer of HD-DCs that lacked the IL-4Rα did not ameliorate DNBS-induced colitis to any significant degree. Given that lack of IL-4Rα does not affect the DCs ability to present antigen^21^, we surmise that IL-4 signaling is of direct importance to the HD-DC regulatory program. The use of IL-4Rα^−/−^ HD-DCs highlighted the reciprocity in the regulation of IL-4 and IL-10 production and the inhibition of colitis, since splenocytes from mice given these cells displayed no increase in IL-4 or IL-10. Interaction between parasite molecules and host factors in the regulation of host cell function is not unprecedented: for example, helminth-products and host antibodies cooperated to enhance macrophage-myofibroblast communication that mediated intestinal wound repair^58^.

**Figure 8:**
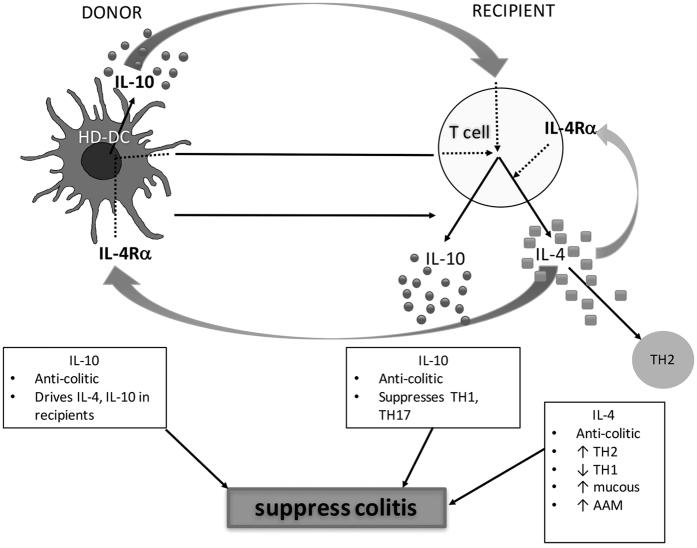
Summary model of the role of IL-4 signaling in HD-DC suppression of colitis. Recipient IL-4Rα signaling is required for HD-DC induction of IL-4, and suppression of colitis. HD-DCs induce IL-4 production in recipients, which likely enhances recipient IL-4 production via autocrine signaling through IL-4Rα. Activation of IL-4Rα is required to drive the regulatory anti-colitic phenotype of HD-DCs, in addition to splenic IL-4 and IL-10 in recipient mice. As HD-DCs are not a source of IL-4, HD-DC induced recipient IL-4 is the likely source for IL-4Rα activation on HD-DCs. HD-DCs produce IL-10 *in vivo*, and drive synthesis of IL-4 and IL-10 in recipient mice. Overall, IL-10 is an anti-colitic cytokine that can suppress TH1 and TH17 pro-inflammatory responses, and is required to establish Th2 immune responses; IL-4 is also anti-colitic, driving TH2 and suppressing TH1 immune responses, driving the alternative activation of macrophages, and enhancing mucus production.

In summary, using the DNBS model of colitis that recapitulates some aspects of human inflammatory bowel disease, and considering cellular immunotherapy as a novel facet of helminth therapy for inflammatory disease, the data obtained support three conclusions: (1) HD-DCs, but not IL-4 educated AADCs, block DNBS-induced colitis; (2) the reduced severity of DNBS-induced colitis due to adoptive transfer of HD-DCs is dependent on IL-4α signaling in both the recipient mice and in the HD-DCs, indicating that the worm antigen synergizes with host IL-4 to elicit a regulatory program in the DCs; and, (3) cross-talk between IL-4 and IL-10 is required for the anti-colitic effect of the HD-DCs (Fig. 8). Much remains to be done to define the effector mechanisms that underlie the HD-DC inhibition of colitis. However, the need for IL-4Rα-IL-4 signaling in the donor cells and in the recipients adds to awareness of the complexity of the *in vivo* events needed for the HD-DC to be effective, and that would likely be essential in patients identified as possible candidates for this novel therapeutic approach.

## Methods

### Animals

Animal experiments were performed in accordance with the Canadian Council on Animal Welfare, under a protocol approved by the University of Calgary Animal Care Committee (protocol AC13–0015). Balb/c mice (Charles River Laboratories), IL-10 KO mice (Jackson Laboratories), and IL-4Rα^−/−^ mice and their WT controls (BALB/c background; gift from Dr. Frank Brombacher) were maintained at the Animal Resources Centre, University of Calgary, with free access to chow and water. Mice were used between 8–12 weeks of age.

### HD antigen

Adult *H. diminuta* (HD) worms were recovered from the small intestine of infected rats, washed thoroughly, homogenized in PBS, and centrifuged (4000 rpm, 30 min, 4 °C). The PBS soluble fraction was removed and centrifuged a second time and protein content determined via Bradford Assay; aliquots of HD antigen were stored at −80 °C. Several batches of HD antigen were used throughout these experiments, all of which were standardized by their ability to suppress LPS-induced TNFα production by THP1 monocytic-like cells (48 hours, 1 μg/mL) by ≥40%^59^. A ToxinSensor Chromogenic LAL kit (Genscript) revealed trace levels (65 pg LPS/mg HD antigen) of endotoxin contamination^20^.

### HD-DC generation and adoptive transfer

Bone Marrow (BM) cells were harvested and seeded at 5 × 10^6^/10 mL in RPMI medium (Sigma Aldrich, St. Louis, MO) supplemented with 10% FBS, 2% Pen/Strep, 1% sodium pyruvate and Glutamax^TM^ (all Gibco, Grand Island, NY), with recombinant murine GM-CSF (20 ng/mL; Peprotech) for differentiation to dendritic cells (DC). Cells were fed an additional 10 mL of supplemented medium and 20 ng/mL of GM-SCF on Day 3, and another 5 mL of medium with 40 ng/mL GM-SCF on day 6. On Day 8, floating and loosely adherent cells were removed by gentle pipetting. Cells were 91 ± 2% CD11c^+^ as determined by flow cytometry. Cells were washed, resuspended in 10% DMSO in FBS (5–10 × 10^6^/mL) and stored in liquid nitrogen. For *in vitro* experiments, cells were treated with HD antigen (100 μg/mL), 24 h prior to exposure of LPS (1 μg/mL) ± IL-4 (20 ng/mL); supernatants were collected 24 h later. For adoptive transfer experiments, cells were left untreated, treated with IL-4 (20 ng/mL), with HD extract (100 μg/mL), or both for 24 hours; doses of IL-4 and HD were selected based on previously published methods for AADC generation, and immunomodulation of THP-1 cells, respectively^20^,^21^,^59^. Cells were washed (x3) with sterile PBS, and 1 × 10^6^ cells in 500 μL of sterile PBS injected intraperitoneally (i.p.), 48 h prior to dinitrobenzene sulfonic acid (DNBS) challenge.

### Induction of Colitis

Mice were anesthetized (inhaled isoflurane (4%)) and colitis induced by intra-rectal (i.r.) instillation of 3 mg DNBS (MP Biomedicals, Santa Barbara, CA) dissolved in 100 μl of 50% ethanol (PBS as negative control) using a polyethylene catheter inserted approximately 3 cm from the anus^7^.

### Assessment of Colitis

Mice were examined daily for signs of disease and weight change. Mice were euthanized 72 h after the induction of colitis, and the colon was removed for assessment and a macroscopic damage score was calculated as previously published: presence of visceral adhesions (0–2 points); colonic shortening (relative to controls, multiplied by 0.1), proportion of colonic inflammation (% inflamed regions multiplied by 0.05), diarrhea, fecal blood, and erythema (0–1 point each)^20^. Proportion of colon shortening and colon inflammation were multiplied by factors to produce a weighted value that represents approximately 20% of the total severity of macroscopic disease observed in the DNBS model of colitis, based on our previous observations of the positive control group (e.g. The average colonic shortening of DNBS treated animals is approximately 25%, so multiplying by 0.1 gives 2.5 points, or approximately 20% of the damage score, assuming an approximate damage score of 12 points). Weight change was not included in the macroscopic disease score, as our intention was to focus on colon pathology. A 1 cm portion of the colon, measured 20% from the distal end, was fixed in formalin, embedded in paraffin, sectioned (5 μm) and stained with haematoxylin and eosin (H&E). Histopathology was assessed in a blinded fashion using a 12-point scale; cellular infiltration (0–3 points); loss of architecture (0–3 points); muscle thickening (0–2 points); goblet cell depletion, crypt abscess, edema, and ulcers (0–1 point each)^7^.

### Real Time PCR

RNA from cells was isolated with TRIzol reagent (Invitrogen) following the manufacturer’s instructions, quantified with a Nanodrop Spectrophotometer, and used to generate cDNA with the iScript kit (Bio-Rad Laboratories, Mississauga, ON, Canada). PCR primers were created using the NCBI Primer-BLAST program, and synthesized by Invitrogen. Primers used are listed in Table 1.

### Flow Cytometry

Splenocytes (5 × 10^6^/mL) were stimulated with plate-bound anti-CD3 antibodies (2 μg/mL) and soluble anti-CD28 antibodies (2 μg/mL) for 3 days, and brefeldin-A (5 μg/mL: Biolegend) for the final 16 h of culture. Cells were resuspended in flow buffer (PBS with 0.1% sodium azide and 1% FBS), and stained with anti-CD16/32 TruStain fcX (1: 200, Biolegend) and a live/dead viability dye (BV-510 at 1:500; BD Biosciences) for 30 min at 4 °C. Staining of surface markers (APC-CD4, FITC-CD8α, PercpCy5.5-CD19, all Biolegend) was performed for 30 min at 4 °C in the dark. Cells were fixed, permeabilized using the FIX/PERM set (Biolegend) and blocked in 5% rat serum for 10 min at room temperature prior to intracellular staining for IL-4 with PE-Cy7-anti-IL-4 (Biolegend) for 20 min at room temperature. Data was analyzed using BD FACSDIVA software (BD Biosciences, San Jose, CA, USA).

### Western Blotting

Cells were lyzed in modified radioimmunoprecipitation assay (RIPA) buffer containing 50 mM Tris HCL pH 8, 150 mM NaCl, 1% NP-40, 0.5% sodium deoxycholate and 0.1% SDS, and protein content measured. Protein samples (40 μg) were boiled with loading dye, loaded on a 12% SDS-PAGE gel, transferred to a nitrocellulose membrane, and blocked with 5% nonfat milk in PBS. Samples were probed for β-actin (anti-goat, Santa Cruz Biotechnology, Santa Cruz, CA), arginase-1 (anti-rabbit, Santa Cruz), and Ym-1 (anti rabbit, Stem Cell Technologies, Vancouver BC, Canada) at concentrations of 1:1000, 1:200, and 1:500 respectively. Following incubation with horseradish peroxidase-conjugated secondary antibodies (1:1000–2000), membranes were washed in tris-buffered saline with 1% tween-20, stripped using 100 mM β-mercaptoethanol buffer for 10 min at 50 °C. Blots were developed and densitometry accomplished using Gel Capture MicroChemi Chemiluminescence Substrate.

### Cytokine analysis

Cytokine levels were assessed in the supernatants of a) BMDCs left untreated or treated with HD antigens (100 μg/mL, 24 h) and b) splenocytes (5 × 10^6^) stimulated with concanavalin A (2 μg/mL) for 48 h. ELISAs were performed using DuoSet® kits from R&D Systems (Minneapolis, MN), following the manufacturers’ instructions.

### Statistical Analysis

All data are displayed as mean ± SEM; p < 0.05 was accepted as statistically significant. A D’Agonstino Pearson omnibus normality test was used to assess a Gaussian distribution. For parametric data, statistical outliers were removed using the Grubb’s test, and a one-way ANOVA with Tukey’s post-test, or Sidak’s multiple comparison test was performed to assess significance between groups. For repeated measure ANOVAs, a Dunnett’s multiple comparison post-test was performed. When comparisons between two groups were being made, a two-tailed Student’s t-test was used, and paired where appropriate; for comparison to a mean with a known value, a one-sided, two-tailed t-test was performed. Significant differences were assessed for non-parametric data using a Mann-Whitney or Kruskal-Wallis test with Dunn’s post-test. A Spearman rank correlation was used to assess correlations between non parametric data. For macroscopic and histology damage scores, analysis were conducted on all DNBS treated groups; all other analysis included the negative control. Analyses were conducted with GraphPad Prism 6.0 (GraphPad Software, La Jolla, CA).

## Additional Information

**How to cite this article**: Matisz, C. E. *et al*. Suppression of colitis by adoptive transfer of helminth antigen-treated dendritic cells requires interleukin-4 receptor-α signaling. *Sci. Rep.* **7**, 40631; doi: 10.1038/srep40631 (2017).

**Publisher's note:** Springer Nature remains neutral with regard to jurisdictional claims in published maps and institutional affiliations.

## Figures and Tables

**Table 1:** List of Primers used.

Gene	Forward	Reverse
18 s	GTAGTTGAACCCCATT	CGGTAGTAGCG
arg-1	GTCTGTGGGGCCAAT	GATGCTTCCAACTGCCAGAGC
retnla	GGAACTTCTTGCCAATCCAGC	AAGCACACCCAGTAGCAGTC
chil-3	GTACCCTGGGTCTCGAGGAA	CCTTGGAATGTGCTTTCTCCACAG
